# Molecular Epidemiology of EGFR Mutations in Asian Patients with Advanced Non-Small-Cell Lung Cancer of Adenocarcinoma Histology – Mainland China Subset Analysis of the PIONEER study

**DOI:** 10.1371/journal.pone.0143515

**Published:** 2015-11-23

**Authors:** Yuankai Shi, Junling Li, Shucai Zhang, Mengzhao Wang, Shujun Yang, Ning Li, Gang Wu, Wei Liu, Guoqing Liao, Kaican Cai, Liang’an Chen, Meizhen Zheng, Ping Yu, Xiuwen Wang, Yunpeng Liu, Qisen Guo, Ligong Nie, Jiwei Liu, Xiaohong Han

**Affiliations:** 1 Department of Medical Oncology, Cancer Hospital, Chinese Academy of Medical Sciences & Peking Union Medical College; Beijing Key Laboratory of Clinical Study on Anticancer Molecular Targeted Drugs, No.17 Panjiayuan Nanli, Chaoyang District, Beijing (100021), China; 2 Department of Medical Oncology, Beijing Chest Hospital, Capital Medical University, Beijing (1011429), China; 3 Department of Respiration Medicine, Peking Union Medical College Hospital, Chinese Academy of Medical Sciences & Peking Union Medical College, Beijing (100005), China; 4 Department of Medical Oncology, Henan Cancer Hospital, Zhengzhou (450008), Henan Province, China; 5 Department of Medical Oncology, Guangzhou Chest Hospital, Guangzhou (510095), Guangdong Province, China; 6 Cancer Center, Union Hospital, Tongji Medical College HuaZhong University of Science and Technology, Wuhan (430030), Hubei Province, China; 7 Department of Medical Oncology, Fourth Hospital of Hebei Medical University, Shijiazhuang (050035), Hebei Province, China; 8 Department of Medical Oncology, The 309th Hospital of Chinese People’s Liberation Army, Beijing (100091), China; 9 Department of Medical Oncology, Nanfang Hospital, Southern Medical University, Guangzhou (510515), Guangdong Province, China; 10 Department of Respiration Medicine, Chinese People’s Liberation Army General Hospital, Beijing (100853), China; 11 Department of Medical Oncology, Liaoning Cancer Hospital, Shenyang (110042), Liaoning Province, China; 12 Department of Medical Oncology, Sichuan Cancer Hospital, Chengdu (610041), Sichuan Province, China; 13 Department of Medical Oncology, Qilu Hospital, Shandong University, Jinan (250012), Shandong Province, China; 14 Department of Medical Oncology, The First Hospital of China Medical University, Shenyang (110001), Liaoning Province, China; 15 Department of Medical Oncology, Shandong Cancer Hospital, Jinan (250117), Shandong Province, China; 16 Department of Respiration Medicine, Peking University First Hospital, Beijing (100034), China; 17 Department of Medical Oncology, The First Affiliated Hospital of Dalian Medical University, Dalian (116011), Liaoning Province, China; University of Central Florida, UNITED STATES

## Abstract

Epidermal growth factor receptor (EGFR) mutations are the strongest response predictors to EGFR tyrosine kinase inhibitors (TKI) therapy, but knowledge of the EGFR mutation frequency on lung adenocarcinoma is still limited to retrospective studies. The PIONEER study (NCT01185314) is a prospective molecular epidemiology study in Asian patients with newly diagnosed advanced lung adenocarcinoma, aiming to prospectively analyze EGFR mutation status in IIIB/IV treatment-naïve lung adenocarcinomas in Asia. We report the mainland China subset results. Eligible patients (≥20 yrs old, IIIB/IV adenocarcinoma and treatment-naïve) were registered in 17 hospitals in mainland China. EGFR was tested for mutations by amplification refractory mutation system using biopsy samples. Demographic and clinical characteristics were collected for subgroup analyses. A total of 747 patients were registered. Successful EGFR mutation analysis was performed in 741, with an overall mutation rate of 50.2%. The EGFR active mutation rate is 48.0% (with 1.3% of combined active and resistance mutations). Tobacco use (>30 pack-year vs. 0–10 pack-year, OR 0.27, 95%CI: 0.17–0.42) and regional lymph nodes involvement (N3 vs. N0, OR 0.47, 95%CI: 0.29–0.76) were independent predictors of EGFR mutation in multivariate analysis. However, even in regular smokers, the EGFR mutation frequency was 35.3%. The EGFR mutation frequency was similar between diverse biopsy sites and techniques. The overall EGFR mutation frequency of the mainland China subset was 50.2%, independently associated with the intensity of tobacco use and regional lymph nodes involvement. The relatively high frequency of EGFR mutations in the mainland China subset suggest that any effort to obtain tissue sample for EGFR mutation testing should be encouraged.

## Introduction

Lung cancer is the leading cause of cancer-related death in the world [1]. Platinum-based chemotherapy remain the main treatment choice for advanced non-small-cell lung cancer (NSCLC) [2]. However, epidermal growth factor receptor (EGFR) tyrosine kinase inhibitors (TKI) therapy recently achieved promising successes in NSCLC patients harboring EGFR active mutations [3–5], significantly prolonging patients’ survival [6,7].

Therefore, it is of great importance to determine the prevalence of EGFR mutations frequency. Studies in different populations identified some subgroups (adenocarcinoma histology, women, never-smokers and East Asian ethnic origin) with higher EGFR active mutation rate [8,9]. However, several retrospective studies in resectable and advanced lung adenocarcinoma have suggested that gender was not an independent factor for EGFR mutation [10,11]. Therefore, it is significant to identify demographic and clinical characteristics associated with EGFR mutations, to allow identifying subpopulations of patients at high risk of harboring EGFR mutation, thus allowing the oncologists to decide which first-line treatment may offer the most benefits.

Most studies assessed EGFR mutation status in a clinical trial setting, or retrospectively based on archival tissue samples which might not sufficiently comply with real-life practice on an epidemiological point of view. Therefore, prospective studies of EGFR mutation status in clinical settings are needed.

A prospective epidemiological study has been conducted in Spain to screen for EGFR mutations in lung cancer [12], but no prospective studies were carried out for Asian patients. The PIONEER study (NCT01185314) is a large prospective molecular epidemiology study in Asian patients with newly diagnosed advanced lung adenocarcinoma, aiming to assess their EGFR mutation status [13]. This study enrolled a total of 1510 patients in 51 investigational sites in 7 Asian countries/regions (mainland China, Hong Kong, India, Philippines, Taiwan, Thailand and Vietnam) to investigate EGFR mutation frequency and to correlate these mutations with patients’ demographic and clinical characteristics, as well as with various tissue sampling techniques. In the present report, we present the results for the subset of patients from mainland China.

## Material and Methods

### Study Design and Patients

The PIONEER study (NCT01185314) is an epidemiological, multicenter, open-label and non-comparative study of EGFR mutation status in patients with newly diagnosed advanced (stage IIIb/IV) NSCLC.

Eligibility criteria were: 1) ≥20 years old; 2) histologically or cytologically confirmed advanced lung adenocarcinoma (stage IIIb/IV); and 3) treatment-naïve. Patients were registered in 17 hospitals in mainland China.

The study was performed according to the Declaration of Helsinki and good clinical practice guidelines. The study was approved by the Ethics Committees of all study centers. All patients provided a written informed consent for participation in the study and the use of tumor samples (tissue or cytology).

### Data collection

Demographic data of patients were collected, including gender, age, and smoking status. Disease data included date of first NSCLC diagnosis, histological type, AJCC stage, nodal status, and distant metastases. Standardized case report forms were used to record the data in accordance with the protocol’s instructions. Smoking was assessed using two ways. First, patients were classified according to their actual smoking status (never-smoked means that the subject smoked no cigarettes during his entire lifetime; ex-smoker means that the subject no longer smokes; occasional smoker means that the subject smokes, but not every day; and regular smoker means that the subject smokes every day). Second, smoking patients were classified according to their tobacco consumption, in pack-year.

### EGFR Mutation Analysis

Tumor samples were obtained from primary or metastatic lesions, and were handled and stored following laboratories' quality control requirements. Biopsy site and technique were recorded. Cytological samples were accepted only when histological material was unavailable. After tumor DNA extraction, EGFR mutation was analyzed at Laboratory of Medical Oncology, Cancer Hospital, Chinese Academy of Medical Sciences & Peking Union Medical College; Beijing Key Laboratory of Clinical Study on Anticancer Molecular Targeted Drugs by an amplification refractory mutation system (ARMS)-based EGFR mutation detection kit (EGFR RGQ PCR kit, Qiagen, Crawley, UK). This kit allows the detection of 29 mutations in the EGFR gene.

### Statistical Analyses

Statistical analysis was performed by the Statistics and Programming department of Astra Zeneca Japan using SAS 8.2 (SAS Institute, Cary, NY, USA). The per protocol analysis (PPS) set was used for all statistical analyses. Mutations prevalence and corresponding 95% confidence intervals (95%CI) were calculated using the Wilson score method. Associations between mutations and demographic and clinical characteristics were analyzed by χ2 tests or Fisher’s exact tests, as appropriate. Characteristics associated with mutations with a P-value <0.05 were included in a multivariate logistic model. All analyses were two-sided and a P-value <0.05 was considered significant. In the multivariate analysis, a P-value <0.01 was considered significant.

## Results

### Baseline characteristics of the study population

From September 2010 to July 2011, a total of 747 patients (747/1510, 49.5% of the overall study population) from 17 hospitals in mainland China were registered for analysis (PPS). Among them, 397 (53.1%) were men and 350 (46.9%) were women. The mean age at registration was 57.4±11.4 years, ranging from 20 to 83 years. All patients were Chinese population. The total number of never-smokers was 421 (56.4%), ex-smokers were 136 (18.2%), occasional smokers were 19 (2.5%) and regular smoker were 171 (22.9%). Among patients with a positive smoking history (n = 324), the mean pack-years was 32.9±24.0. For 97.2% (n = 726) of patients, the time interval between original diagnosis and enrolment into the study was <6 months. At original diagnosis, distant metastases were found in 615 (82.3%) patients, and 132 (17.7%) patients suffered from a locally advanced disease. The most common site for biopsy was lung (549 patients), local lymph nodes (77 patients) and distant lymph nodes (47 patients). The most common method of biopsy was image-guided core biopsy (222/747, 29.7%), bronchoscopic biopsy (180/747, 24.1%) and incisional biopsy (95/747, 12.7%). In the majority of patients, tissue sample was taken from the primary tumor (n = 541, 72.4%) and was fixed in 10% neutral buffered formalin (n = 639, 85.5%).

### Factors associated with EGFR mutation

**Fig 1:**
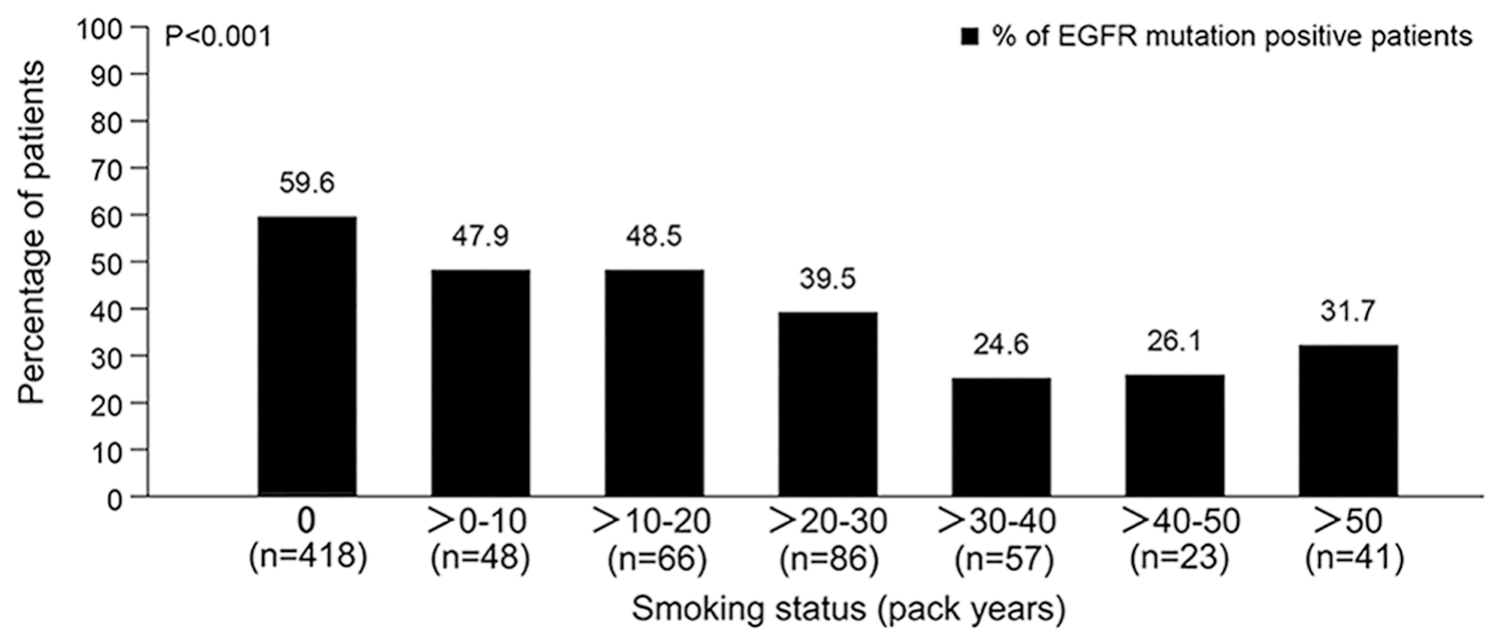
EGFR mutation frequency according to tobacco consumption (in pack-years).

In the PPS population, EGFR mutation analysis was successful in 741 patients (99.2%). Among the 741 evaluable patients, 372 (50.2%, 95%CI: 46.6–53.8) were EGFR mutation-positive (M+), and 369 (49.8%, 95%CI: 46.2–53.4) were EGFR mutation-negative (M-). In univariate analyses, as expected, the female gender was significantly associated with higher EGFR mutation frequency (60.6% for female vs. 41.0% for male, P<0.001), and smoking status was associated with M+, with a higher incidence of EGFR M+ in patients who never smoked (59.6%, P<0.001) (Fig 1) or those who smoked 0–10 pack-years (58.4%, P<0.001) (Table 1). As smoking and pack-years were covariates, only the pack-year variable was included in the multivariate analysis. Stage classification (53.0% for stage IV vs. 38.6% for stage IIIb, P = 0.002) and regional lymph node involvement (62.1% for N0, 52.6% for N1-2 and 43.8% for N3, P = 0.009) were also factors associated with EGFR mutation frequency in univariate analyses.

**Table 1:** Subgroups analysis for EGFR mutation testing (PPS).

Subgroup		Positive			Negative			p-value
	N	N	%	95% CI	n	%	95% CI	
China	741	372	50.2	46.6–53.8	369	49.8	46.2–53.4	
Age group								
<65	540	274	50.7	46.5–54.9	266	49.3	45.1–53.5	0.810
65–74.9	149	74	49.7	41.7–57.6	75	50.3	42.4–58.3	
>75	52	24	46.2	33.3–59.5	28	53.8	40.5–66.7	
Gender								
Men	393	161	41.0	36.2–45.9	232	59.0	54.1–63.8	<0.001
Women	348	211	60.6	55.4–65.6	137	39.4	34.4–44.6	
Smoking								
Never	418	249	59.6	54.8–64.2	169	40.4	35.8–45.2	<0.001
Ex	134	54	40.3	32.4–48.8	80	59.7	51.2–67.6	
Occasional	19	9	47.4	27.3–68.3	10	52.6	31.7–72.7	
Regular	170	60	35.3	28.5–42.7	110	64.7	57.3–71.5	
Pack years								
0–10	466	272	58.4	53.8–62.8	194	41.6	37.2–46.2	<0.001
10–30	152	66	43.4	35.8–51.4	86	56.6	48.6–64.2	
>30	121	33	27.3	20.1–35.8	88	72.7	64.2–79.9	
Time from original diagnosis								
<6 months	720	361	50.1	46.5–53.8	359	49.9	46.2–53.5	0.949
6–12 months	12	6	50.0	25.4–74.6	6	50.0	25.4–74.6	
>12 months	9	5	55.6	26.7–81.1	4	44.4	18.9–73.3	
Malignant pleural effusion								
Absent	563	276	49.0	44.9–53.1	287	51.0	46.9–55.1	0.253
Present	178	96	53.9	46.6–61.1	82	46.1	38.9–53.4	
Primary tumour								
T1	75	35	46.7	35.8–57.8	40	53.3	42.2–64.2	0.230
T2	229	122	53.3	46.8–59.6	107	46.7	40.4–53.2	
T3	110	48	43.6	34.7–53.0	62	56.4	47.0–65.3	
T4	287	142	49.5	43.7–55.2	145	50.5	44.8–56.3	
TX	40	25	62.5	47.0–75.8	15	37.5	24.2–53.0	
Regional lymph nodes								
N0	95	59	62.1	52.1–71.2	36	37.9	28.8–47.9	0.009
N1-2	340	179	52.6	47.3–57.9	161	47.4	42.1–52.7	
N3	292	128	43.8	38.3–49.6	164	56.2	50.4–61.7	
NX	13	5	38.5	17.7–64.5	8	61.5	35.5–82.3	
Stage classification								
IIIB	145	56	38.6	31.1–46.7	89	61.4	53.3–68.9	0.002
IV	596	316	53.0	49.0–57.0	280	47.0	43.0–51.0	
Tumour grade								
I	49	19	38.8	26.4–52.8	30	61.2	47.2–73.6	0.047
II	138	63	45.7	37.6–54.0	75	54.3	46.0–62.4	
III	226	115	50.9	44.4–57.3	111	49.1	42.7–55.6	
IV	10	2	20.0	5.7–51.0	8	80.0	49.0–94.3	
X	318	173	54.4	48.9–59.8	145	45.6	40.2–51.1	

Never-smoked means that the subject smoked no cigarettes during his entire lifetime; ex-smoker means that the subject no longer smokes; occasional smoker means that the subject smokes, but not every day; and regular smoker means that the subject smokes every day.

Age, time from original diagnosis, malignant pleural effusion, and primary tumor were not associated with M+ status. Tumor grade seemed to be significantly associated with EGFR mutation frequency; however, this variable was documented for a little number of patients, mainly due to the limited amount of tissue available for diagnosis, and was therefore not included in the multivariate analysis. The distribution of EGFR M+ according to biopsy site, type and technique was relatively similar (S1 Table). Patients with lymph nodes biopsy didn’t show lower EGFR mutation frequency compared with other biopsy sites (47.2% vs 50.8%, *P* = 0.4591).

Multivariate analysis identified pack-year and regional lymph node involvement as independent predictors of EGFR M+. Patients smoking 10–30 (OR 0.54, 95%CI: 0.37–0.78) or more than 30 (OR 0.27, 95%CI: 0.17–0.42) pack-year had less chance to harbor an EGFR mutation than those smoking less than 10 pack-year. Tumors with regional lymph nodes involvement (stage N1-2, OR 0.71, 95%CI: 0.44–1.14; and N3, OR 0.47, 95%CI: 0.29–0.76) were also less likely to harbor EGFR mutations than patients without lymph nodes involvement (N0) (Table 2).

**Table 2:** Multivariate analysis using logistic model for EGFR mutation testing (PPS).

	OR	95% CI		P
Pack years				
0–10	1.00			
10–30	0.54	0.37	0.78	<0.001
>30	0.27	0.17	0.42	
Regional lymph nodes				
N0	1.00			
N1-2	0.71	0.44	1.14	0.003
N3	0.47	0.29	0.76	

OR: odds ratio; CI: confidence interval

For patients who provided both histology and cytology samples, test results from histology samples were used.

The significance level for variable selection was 0.01.

NX in regional lymph nodes were excluded from the analysis.

The number of patients used for this analysis was 725.

### Types of EGFR mutation

The most common mutations were exon 19 deletion and L858R point mutation, observed in 182 (24.6%) and 169 (22.8%) patients, respectively. Out of the 372 EGFR M+ patients, 346 patients (46.7%) had EGFR active mutations alone, 16 patients (2.2%) had resistant mutations alone, and 10 patients (1.3%) had both active and resistant EGFR mutations (Table 3). Pack-year and regional lymph nodes involvement were also identified as independent factors significantly associated with EGFR active mutations by univariate and multivariate analysis (S2 Table and S3 Table).

**Table 3:** Summary of individual mutation types including multiple mutations.

	n	%
Number of patients with EGFR mutation test success	741	100.0
Active mutations alone	346	46.7
G719X	2	0.3
G719X, L861Q	1	0.1
Deletion	176	23.8
Deletion, L858R	3	0.4
L858R	159	21.5
L861Q	5	0.7
Resistance mutations	16	2.2
S768I	6	0.8
Exon 20 other (Insertion)	10	1.3
Combination of active and resistance mutations	10	1.3
G719X, S768I	1	0.1
Deletion, S768I, L858R	1	0.1
Deletion, Exon 20 other (Insertion)	2	0.3
T790M, L858R	3	0.4
L858R, Exon 20 other (Insertion)	3	0.4
Negative	369	49.8

Additionally, nine patients provided both histology and cytology samples, among which one patient had mutation results that did not match.

## Discussion

EGFR mutations are currently the strongest response predictor to EGFR-TKI therapy [14]. In addition, with the establishment of first-line treatment using EGFR-TKI [6,7], the knowledge of EGFR mutation status in treatment-naïve advanced NSCLC, especially lung adenocarcinoma, is of great importance for clinical decision. Previous data were generally derived from retrospective studies and clinical trials, which might not reflect real-life. PIONEER is a large epidemiological study prospectively investigating the EGFR mutation status in Asian treatment-naïve advanced NSCLC patients [13], and we report the results from the mainland China subset.

In our study (PPS population), the overall EGFR mutation frequency was 50.2%, similar to previous studies in East Asian patients [6,15], but much higher than in Caucasian lung adenocarcinoma patients [12,16]. Besides a different ethnicity distribution, EGFR mutations are also suggested to be more common in adenocarcinoma, women, and never-smokers [8,9]. Rekhtman et al. reported that “pure” squamous cell lung cancer, verified by immunohistochemistry as Np63 (+)/TTF-1(-), lacked EGFR mutations [17], thus leaving lung adenocarcinoma as the major candidate for EGFR screening in NSCLC. In the present prospective study, we also found that the impact of gender was not significant after adjustment for smoking status, and did not have any interaction with smoking (data not shown).

Smoking status was identified as an independent predictor of EGFR mutation in the present study. We observed that the EGFR mutation incidence decreased with the increasing tobacco consumption, which is similar to a retrospective by Li et al. in resectable NSCLC [18]. When smoking was classified into never-smoked, ex-smoker, occasional smoker, and regular smoker according to their smoking frequency, there was a wide gap between never-smoked and ever-smoked patients. However, even in regular smokers, EGFR mutation was still found in 35.3% patients, indicating the necessity of EGFR mutation testing in this group of patients.

Another interesting finding of the present study was that EGFR mutation frequency independently decreased with the involvement of regional lymph node, and patients with N3 disease had the least chance of harboring an EGFR mutation. This finding is supported by a study by Na et al. in a Korean population (OR 0.2, 95%CI: 0.0–0.7, P = 0.02) [19]. There is little chance that this observation might be due to the tumor sample volume because the ARMS approach has a well-known detection sensitivity, and the EGFR M+ frequency according to biopsy sites was comparable. The plausible explanation still warrants further investigations.

EGFR T790M mutation is associated with shorter progression-free survival with EGFR-TKI therapy in patients with NSCLC [20,21]. However, the prevalence of the EGFR T790M mutation varied by population and technique. Su et al. detected T790M in 2.8% of Chinese patients with TKI-naïve NSCLCs [20], while Rosell et al., using a Taqman assay, reported that 35% of EGFR M+ (L858R or 19 del) NSCLC had concomitant T790M mutation in Caucasians [21]. In this prospective cohort of treatment-naïve advanced lung adenocarcinoma, the T790M mutation was found in only 0.4% of our Chinese patients using the ARMS assay, accounting for 0.8% of EGFR M+ patients, all of which were observed concomitantly with the EGFR L858R mutation. This rate of the T790M mutation is much lower than the frequency observed by Su et al. (2.8%) [20]. This might be due to the smaller sample size in their study, and by the different detection method. The EGFR exon 20 insertion mutation was also suggested to induce a poor responses to EGFR-TKI [22], and was reported in about 2% of NSCLC or lung adenocarcinoma [23–25], which is similar to our report (2.0%).

The present study has some limitations. Indeed, despite all our efforts, we were unable to recruit all lung cancer patients, mainly because some of them did not consent, which could introduce a bias. Furthermore, tobacco consumption was self-reported, and might suffer from some under-reporting.

## Conclusion

In conclusion, from the PIONEER prospective study, the overall EGFR mutation frequency of the mainland China subset was 50.2%. The EGFR active mutation rate is 48.0% (with 1.3% of combined active and resistance mutations). The relatively high frequency of EGFR mutation in this population and the comparable EGFR M+ rate among diverse biopsy sites and techniques suggest that any effort to gain tissue samples for EGFR mutation testing should be encouraged.

## Supporting Information

S1 Table Overall analysis of EGFR mutation test by sample type (PPS). (DOC)

S2 Table Subgroup analysis for EGFR active mutations alone (PPS). (DOC)

S3 Table Logistic model analysis for EGFR active mutations alone (PPS). (DOC)
