# Supplementary material for: Molecular Epidemiology of EGFR Mutations in Asian Patients with Advanced Non-Small-Cell Lung Cancer of Adenocarcinoma Histology – Mainland China Subset Analysis of the PIONEER study
Source: PLoS One. 2015 Nov 23;10(11):e0143515. doi: 10.1371/journal.pone.0143515 (PMC4657882; doi:10.1371/journal.pone.0143515)
Supplement: S1 Table — (DOC) [file pone.0143515.s001.doc]

**S1 Table .** Overall analysis of EGFR mutation test by sample type (PPS)

|  | | **Positive** | | | **Negative** | | |
| --- | --- | --- | --- | --- | --- | --- | --- |
| Subgroup | N | n | % | 95% CI | n | % | 95% CI |
| China | 741 | 372 | 50.2 | 46.6 - 53.8 | 369 | 49.8 | 46.2 - 53.4 |
| Biopsy site |  |  |  |  |  |  |  |
| Local lymph nodes | 76 | 39 | 51.3 | 40.3 - 62.2 | 37 | 48.7 | 37.8 - 59.7 |
| Distant lymph nodes | 47 | 19 | 40.4 | 27.6 - 54.7 | 28 | 59.6 | 45.3 - 72.4 |
| Lung | 545 | 271 | 49.7 | 45.5 - 53.9 | 274 | 50.3 | 46.1 - 54.5 |
| Pleura | 15 | 9 | 60.0 | 35.7 - 80.2 | 6 | 40.0 | 19.8 - 64.3 |
| Muscle | 3 | 3 | 100.0 | 43.9 - 100.0 | 0 | 0.0 | 0.0 - 56.1 |
| Pleural effusion | 19 | 10 | 52.6 | 31.7 - 72.7 | 9 | 47.4 | 27.3 - 68.3 |
| Others | 36 | 21 | 58.3 | 42.2 - 72.9 | 15 | 41.7 | 27.1 - 57.8 |
| Biopsy type |  |  |  |  |  |  |  |
| Cytology | 19 | 10 | 52.6 | 31.7 - 72.7 | 9 | 47.4 | 27.3 - 68.3 |
| Percutaneous core biopsy | 20 | 12 | 60.0 | 38.7 - 78.1 | 8 | 40.0 | 21.9 - 61.3 |
| Image-guided core biopsy | 218 | 115 | 52.8 | 46.1 - 59.3 | 103 | 47.2 | 40.7 - 53.9 |
| Core-biopsy (NOS) | 5 | 2 | 40.0 | 11.8 - 76.9 | 3 | 60.0 | 23.1 - 88.2 |
| Incisional biopsy | 95 | 40 | 42.1 | 32.7 - 52.2 | 55 | 57.9 | 47.8 - 67.3 |
| Localisation biopsy | 15 | 10 | 66.7 | 41.7 - 84.8 | 5 | 33.3 | 15.2 - 58.3 |
| Segmental excision | 24 | 18 | 75.0 | 55.1 - 88.0 | 6 | 25.0 | 12.0 - 44.9 |
| Needle biopsy | 34 | 15 | 44.1 | 28.9 - 60.5 | 19 | 55.9 | 39.5 - 71.1 |
| Wedge resection | 19 | 5 | 26.3 | 11.8 - 48.8 | 14 | 73.7 | 51.2 - 88.2 |
| Cytology: fine needle aspiration | 2 | 2 | 100.0 | 34.2 - 100.0 | 0 | 0.0 | 0.0 - 65.8 |
| Cytology: bronchial washings | 1 | 0 | 0.0 | 0.0 - 79.3 | 1 | 100.0 | 20.7 - 100.0 |
| Transbronchial | 9 | 4 | 44.4 | 18.9 - 73.3 | 5 | 55.6 | 26.7 - 81.1 |
| Bronchoscopic | 180 | 84 | 46.7 | 39.5 - 53.9 | 96 | 53.3 | 46.1 - 60.5 |
| Segmentectomy | 2 | 1 | 50.0 | 9.5 - 90.5 | 1 | 50.0 | 9.5 - 90.5 |
| Mediastinascopic | 2 | 2 | 100.0 | 34.2 - 100.0 | 0 | 0.0 | 0.0 - 65.8 |
| Pneumonectomy: extra pericardial | 6 | 3 | 50.0 | 18.8 - 81.2 | 3 | 50.0 | 18.8 - 81.2 |
| Pneumonectomy: intra pericardial | 3 | 2 | 66.7 | 20.8 - 93.9 | 1 | 33.3 | 6.1 - 79.2 |
| Lobectomy | 71 | 38 | 53.5 | 42.0 - 64.6 | 33 | 46.5 | 35.4 - 58.0 |
| Others | 16 | 9 | 56.3 | 33.2 - 76.9 | 7 | 43.8 | 23.1 - 66.8 |
| Sample tissue type |  |  |  |  |  |  |  |
| Primary tumour | 537 | 268 | 49.9 | 45.7 - 54.1 | 269 | 50.1 | 45.9 - 54.3 |
| Metastases | 195 | 100 | 51.3 | 44.3 - 58.2 | 95 | 48.7 | 41.8 - 55.7 |
| Others | 9 | 4 | 44.4 | 18.9 - 73.3 | 5 | 55.6 | 26.7 - 81.1 |
| Method of sample processing |  |  |  |  |  |  |  |
| 4% Neutral Buffered Formalin | 102 | 54 | 52.9 | 43.3 - 62.3 | 48 | 47.1 | 37.7 - 56.7 |
| 10% Neutral Buffered Formalin | 633 | 313 | 49.4 | 45.6 - 53.3 | 320 | 50.6 | 46.7 - 54.4 |
| Others | 6 | 5 | 83.3 | 43.6 - 97.0 | 1 | 16.7 | 3.0 - 56.4 |
