# Supplementary material for: Molecular Epidemiology of EGFR Mutations in Asian Patients with Advanced Non-Small-Cell Lung Cancer of Adenocarcinoma Histology – Mainland China Subset Analysis of the PIONEER study
Source: PLoS One. 2015 Nov 23;10(11):e0143515. doi: 10.1371/journal.pone.0143515 (PMC4657882; doi:10.1371/journal.pone.0143515)
Supplement: S2 Table — (DOC) [file pone.0143515.s002.doc]

**S2 Table .** Subgroup analysis for EGFR active mutations alone (PPS)

|  | | **EGFR Active mutations alone** | | | **Negative or including resistance mutations** | | | **P-value** |
| --- | --- | --- | --- | --- | --- | --- | --- | --- |
| **Subgroup** | **N** | **n** | **%** | **95% CI of %** | **n** | **%** | **95% CI of %** |
| China | 741 | 346 | 46.7 | 43.1 - 50.3 | 395 | 53.3 | 49.7 - 56.9 |  |
| **Age group** |  |  |  |  |  |  |  |  |
| <65 | 540 | 254 | 47.0 | 42.9 - 51.3 | 286 | 53.0 | 48.7 - 57.1 | 0.952 |
| 65-74.9 | 149 | 68 | 45.6 | 37.8 - 53.6 | 81 | 54.4 | 46.4 - 62.2 |  |
| >75 | 52 | 24 | 46.2 | 33.3 - 59.5 | 28 | 53.8 | 40.5 - 66.7 |  |
| **Gender** |  |  |  |  |  |  |  |  |
| Men | 393 | 150 | 38.2 | 33.5 - 43.1 | 243 | 61.8 | 56.9 - 66.5 | <0.001 |
| Women | 348 | 196 | 56.3 | 51.1 - 61.4 | 152 | 43.7 | 38.6 - 48.9 |  |
| **Smoking** |  |  |  |  |  |  |  |  |
| Never | 418 | 233 | 55.7 | 50.9 - 60.4 | 185 | 44.3 | 39.6 - 49.1 | <0.001 |
| Ex | 134 | 52 | 38.8 | 31.0 - 47.3 | 82 | 61.2 | 52.7 - 69.0 |  |
| Occasional | 19 | 8 | 42.1 | 23.1 - 63.7 | 11 | 57.9 | 36.3 - 76.9 |  |
| Regular | 170 | 53 | 31.2 | 24.7 - 38.5 | 117 | 68.8 | 61.5 - 75.3 |  |
| **Pack years** |  |  |  |  |  |  |  |  |
| 0-10 | 466 | 256 | 54.9 | 50.4 - 59.4 | 210 | 45.1 | 40.6 - 49.6 | <0.001 |
| 10-30 | 152 | 61 | 40.1 | 32.7 - 48.1 | 91 | 59.9 | 51.9 - 67.3 |  |
| >30 | 121 | 28 | 23.1 | 16.5 - 31.4 | 93 | 76.9 | 68.6 - 83.5 |  |
| **Time from original diagnosis** |  |  |  |  |  |  |  |  |
| <6 mo | 720 | 336 | 46.7 | 43.0 - 50.3 | 384 | 53.3 | 49.7 - 57.0 | 0.965 |
| 6-12 mo | 12 | 6 | 50.0 | 25.4 - 74.6 | 6 | 50.0 | 25.4 - 74.6 |  |
| >12 mo | 9 | 4 | 44.4 | 18.9 - 73.3 | 5 | 55.6 | 26.7 - 81.1 |  |
| **Malignant pleural effusion** |  |  |  |  |  |  |  |  |
| Absent | 563 | 254 | 45.1 | 41.1 - 49.2 | 309 | 54.9 | 50.8 - 58.9 | 0.126 |
| Present | 178 | 92 | 51.7 | 44.4 - 58.9 | 86 | 48.3 | 41.1 - 55.6 |  |
| **Primary tumour** |  |  |  |  |  |  |  |  |
| T1 | 75 | 35 | 46.7 | 35.8 - 57.8 | 40 | 53.3 | 42.2 - 64.2 | 0.462 |
| T2 | 229 | 112 | 48.9 | 42.5 - 55.3 | 117 | 51.1 | 44.7 - 57.5 |  |
| T3 | 110 | 46 | 41.8 | 33.0 - 51.2 | 64 | 58.2 | 48.8 - 67.0 |  |
| T4 | 287 | 130 | 45.3 | 39.6 - 51.1 | 157 | 54.7 | 48.9 - 60.4 |  |
| TX | 40 | 23 | 57.5 | 42.2 - 71.5 | 17 | 42.5 | 28.5 - 57.8 |  |
| **Regional lymph nodes** |  |  |  |  |  |  |  |  |
| N0 | 95 | 57 | 60.0 | 49.9 - 69.3 | 38 | 40.0 | 30.7 - 50.1 | 0.004 |
| N1-2 | 340 | 166 | 48.8 | 43.6 - 54.1 | 174 | 51.2 | 45.9 - 56.4 |  |
| N3 | 292 | 118 | 40.4 | 34.9 - 46.1 | 174 | 59.6 | 53.9 - 65.1 |  |
| NX | 13 | 4 | 30.8 | 12.7 - 57.6 | 9 | 69.2 | 42.4 - 87.3 |  |
| **Stage classification** |  |  |  |  |  |  |  |  |
| IIIB | 145 | 50 | 34.5 | 27.2 - 42.5 | 95 | 65.5 | 57.5 - 72.8 | 0.001 |
| IV | 596 | 296 | 49.7 | 45.7 - 53.7 | 300 | 50.3 | 46.3 - 54.3 |  |
| **Tumour grade** |  |  |  |  |  |  |  |  |
| I | 49 | 18 | 36.7 | 24.7 - 50.7 | 31 | 63.3 | 49.3 - 75.3 | 0.117 |
| II | 138 | 59 | 42.8 | 34.8 - 51.1 | 79 | 57.2 | 48.9 - 65.2 |  |
| III | 226 | 107 | 47.3 | 40.9 - 53.8 | 119 | 52.7 | 46.2 - 59.1 |  |
| IV | 10 | 2 | 20.0 | 5.7 - 51.0 | 8 | 80.0 | 49.0 - 94.3 |  |
| X | 318 | 160 | 50.3 | 44.8 - 55.8 | 158 | 49.7 | 44.2 - 55.2 |  |
