# Supplementary material for: Molecular Epidemiology of EGFR Mutations in Asian Patients with Advanced Non-Small-Cell Lung Cancer of Adenocarcinoma Histology – Mainland China Subset Analysis of the PIONEER study
Source: PLoS One. 2015 Nov 23;10(11):e0143515. doi: 10.1371/journal.pone.0143515 (PMC4657882; doi:10.1371/journal.pone.0143515)
Supplement: S3 Table — (DOC) [file pone.0143515.s003.doc]

**S3 Table.** Logistic model analysis for EGFR active mutations alone (PPS)

|  |  | **OR** | **95% CI** | **P-value** |
| --- | --- | --- | --- | --- |
| **Pack-years** | 0-10 | 1.00 |  |  |
|  | 10-30 | 0.53 | 0.37-.078 | <0.001 |
|  | >30 | 0.24 | 0.15-0.39 |  |
| **Regional lymph nodes** | N0 | 1.00 |  |  |
|  | N1-2 | 0.66 | 0.41-1.07 | 0.002 |
|  | N3 | 0.44 | 0.27-0.72 |  |
